# Circulating tumor cell status monitors the treatment responses in breast cancer patients: a meta-analysis

**DOI:** 10.1038/srep43464

**Published:** 2017-03-24

**Authors:** Wen-Ting Yan, Xiang Cui, Qing Chen, Ya-Fei Li, You-Hong Cui, Yan Wang, Jun Jiang

**Affiliations:** 1 Breast Disease Center, Southwest Hospital, Third Military Medical University, Chongqing 400038, China; 2 Institute of Toxicology, College of Preventive Medicine, Third Military Medical University, Chongqing 400038, China; 3 Department of Epidemiology, College of Preventive Medicine, Third Military Medical University, Chongqing 400038, China; 4 Institute of Pathology and Southwest Cancer Center, Southwest Hospital, Third Military Medical University Chongqing 400038, China

## Abstract

Whether circulating tumor cells (CTCs) can be used as an indicator of treatment response in breast cancer (BC) needs to be clarified. We addressed this issue by a meta-analysis. PubMed, EMBase and Cochrane library databases were searched in June 2016. Effect measures were estimated as pooled risk ratio (RR), odds ratio (OR) or mean difference by fixed- or random-effect models, according to heterogeneity of included studies. In total, 50 studies with 6712 patients were recruited. Overall analysis showed that there was a significant reduction of CTC-positive rate (RR = 0.68, 95% CI: 0.61–0.76, *P* < 0.00001) after treatment. Subgroup analyses revealed that neoadjuvant treatment, adjuvant treatment, metastatic treatment or combination therapy could reduce the CTC-positive rate, but surgery could not; moreover, the reduction was only found in HER2+ or HER2- patients but not in the triple-negative ones. Reduction of CTC-positive rate was associated with lower probability of disease progression (OR = 0.54, 95% CI: 0.33–0.89, *P* = 0.01) and longer overall survival period (mean difference = 11.61 months, 95% CI: 8.63–14.59, *P* < 0.00001) as well as longer progression-free survival period (mean difference = 5.07 months, 95% CI: 2.70–7.44, *P* < 0.0001). These results demonstrate that CTC status can serve as an indicator to monitor the effectiveness of treatments and guide subsequent therapies in BC.

Metastasis is the major cause of cancer-related death in patients with breast cancer (BC)^1^. Despite the improvements in treatment, metastatic relapse may occur in about 30% of BC patients with lymph node-negative axilla and about 50% of BC patients with positive axilla within 5 years^2^. During the process of metastasis, cancer cells shed from primary tumors and migrate to distal sites through the blood or lymphatic system. Those migrating cells found in peripheral blood are known as circulating tumor cells (CTCs). CTCs are the metastatic precursors, which may have potential roles not only in predicting the risk of metastatic relapse and monitoring the treatment efficacy, but also in acting as a therapeutic target for preventing metastasis of cancers, including BC^3^,^4^,^5^,^6^,^7^,^8^,^9^.

Up to now, CTCs have been well studied and are currently being used in the clinical setting^10^. However, there are controversial results about the effectiveness of different treatments to reduce CTCs. For example, Martin M. *et al*. analyzed the change of CTCs in 117 BC patients and observed a substantial decline in CTC-positive rate after chemotherapy^11^, but Rack B. *et al*. conducted a larger perspective study with 2026 BC patients and found that the detection rate of CTCs after chemotherapy (22.1%) was even a bit higher than the baseline condition (21.5%)^12^. In addition, it needs also to be clarified whether molecular subtypes of BC affect CTC status under same treatments^13^. Hence, we conducted a meta-analysis of the published researches with measurement of CTCs before and after treatment in BC patients, and estimated the CTC-reducing effect of the current anti-tumor therapies. The CTC-reducing effects by different treatments were investigated separately in subgroup analyses, so were the effects in patients with different molecular subtypes. Then we also analyzed the relationship between reduction of CTCs and disease progression probability as well as survival period. This study followed the PRISMA criterions.

## Results

### Study characteristics

**Figure 1:**
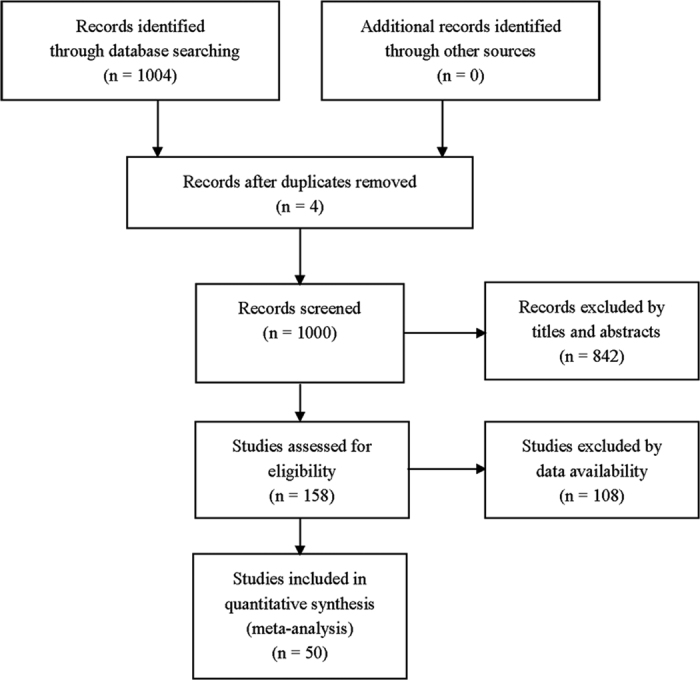
Flowchart for article search.

We initially retrieved 1004 articles through database searching. 846 articles were excluded based on the title and abstract. Further, 108 articles were excluded after reviewing full-texts, in which the results of CTC status before and after treatment were not completely contained, or sample size was less than 20, and or duplicated data with other studies was reported. Finally, 50 studies were eligible for meta-analysis (Fig. 1)^4^,^5^,^9^,^11^,^12^,^13^,^14^,^15^,^16^,^17^,^18^,^19^,^20^,^21^,^22^,^23^,^24^,^25^,^26^,^27^,^28^,^29^,^30^,^31^,^32^,^33^,^34^,^35^,^36^,^37^,^38^,^39^,^40^,^41^,^42^,^43^,^44^,^45^,^46^,^47^,^48^,^49^,^50^,^51^,^52^,^53^,^54^,^55^,^56^,^57^.

The eligible studies were conducted between 2009 and 2016 in America (USA), Asia (China, Japan and Korea) and Europe (Belgium, Czech, Denmark, France, Germany, Greece, Italy, Norway, Russia, Slovakia, Spain, and Turkey). The sample sizes of studies ranged from 21 to 2026 patients, accumulating 8019 patients before treatments and 6712 patients after treatments in total. Some studies reported the results in patients with particular molecular subtype BC, such as the HER2-positve or the triple-negative ones. The treatments performed in the studies included surgery, neoadjuvant treatment, adjuvant treatment, metastatic treatment, etc., either alone or in combination. The main characteristics of these studies are listed in Table S1. The quality of the studies was also estimated (Table S2).

### Overall CTC-positive rate is significantly decreased after therapies

**Figure 2:**
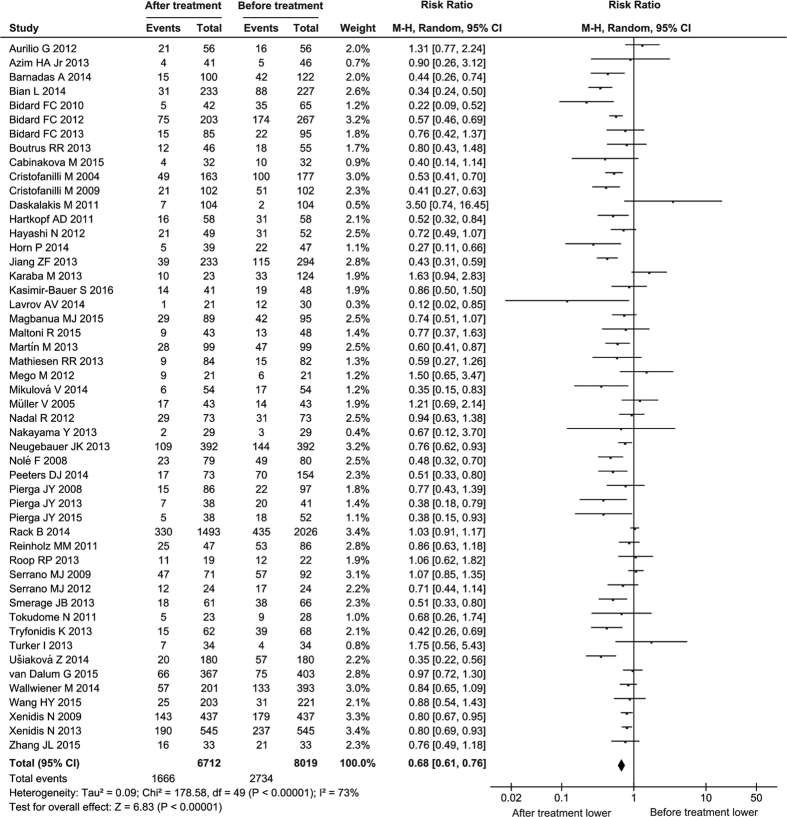
Forest plot for the comparison of CTC-positive rate before and after treatment: overall analysis. The black diamond and its extremities indicate the pooled risk ratio center and 95% confidential interval.

The CTC status was detected with different platforms or methods and presented with different indicators in the 50 studies with 6712 patients. The CTC-positive rate was reported in all of the studies, in which the different cut-off values of CTC count for CTC-capturing methods were used, such as ≥5 CTCs/7.5 mL, ≥1 CTCs/7.5 mL and so on, and the different expression thresholds of epithelial genes (EpCAM, CK18, CK19) were used for RT-PCR method. Some studies also presented the CTC status as CTC count. We first performed an overall analysis of the 50 studies with 6712 patients with CTC-positive rate by the random-effects model, and found that the CTC-positive rate was significantly decreased after treatment compared to the baseline (RR = 0.68, 95% CI: 0.61 to 0.76, *P* < 0.00001; I^2^ = 73%, *P* < 0.00001) (Fig. 2).

**Figure 3:**
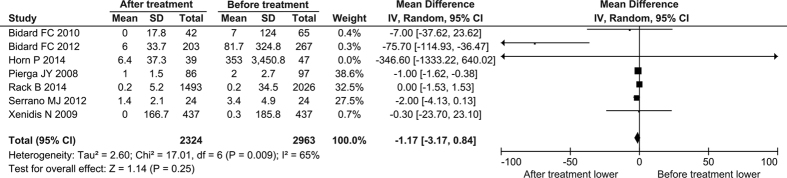
Forest plot for the comparison of CTC count before and after treatment: overall analysis. The black diamond indicates the difference of CTC counts (cells/7.5 mL peripheral blood; the post-therapeutic counts minus the pre-therapeutic counts). Its center indicates the mean and the extremities indicate the 95% confidential interval.

We then analyzed the change of CTC counts in the 7 studies (2324 cases) that simultaneously reported CTC-positive rate and CTC count before and after treatments. No significant change of CTC counts was observed (mean difference = −1.17 CTCs/7.5 mL, 95% CI: −3.17 to 0.84, *P* = 0.25; I^2^ = 65%, *P* = 0.009) (Fig. 3), while a significant reduction was observed with CTC-positive rate in the 7 studies investigated after treatment (RR = 0.65, 95% CI: 0.48 to 0.87, *P* = 0.004; I^2^ = 85%, *P* < 0.00001) (Figure S1). This inconsistent results between CTC-positive rate and CTC count might attribute to the heterogeneity among the studies, because the CTC count, a continuous variable, could be more susceptible to the variance caused by individual study than the CTC-positive rate, a dichotomized variable. So we made a sensitivity analysis by removing each study, and found that one of them (Bidard FC 2012) substantially affected the result. Once it was excluded, the significant decrease of CTC level after treatment was observed compared to pre-treatment (mean difference = −0.94, 95% CI: −1.49 to −0.38, *P* = 0.0010 by fixed-effects model). These results confirmed the promising application of CTCs in monitoring the effectiveness of treatments for BC.

**Figure 4:**
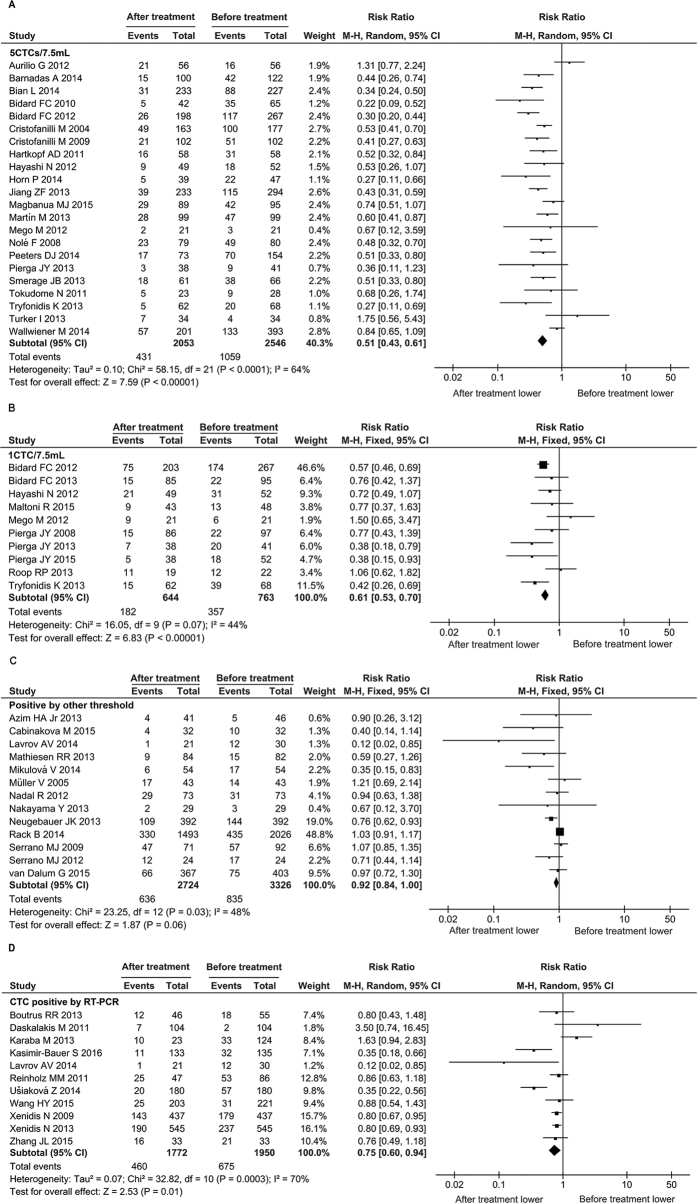
Forest plot for the comparison of CTC-positive rate before and after treatment: subgroup analysis of different CTC determination methods (threshold). The results of ≥5 CTCs/7.5 mL as positive (**A**) ≥1 CTCs/7.5 mL as positive (**B**) other threshold as positive (**C**) and RT-PCR method (**D**) are shown, respectively. The center of black diamond and its extremities indicate the pooled risk ratio and 95% confidential interval.

In addition, different methods as well as different cut-off values for a method were used in the studies. Hence, we also investigated the CTC-positive rate before and after treatment in the subgroups of different CTC-measuring methods. A significant reduction of CTC-positive rate after treatment was observed no matter how the CTC positivity was defined as ≥5CTCs/7.5 mL, ≥1CTCs/7.5 mL, or other threshold including a threshold for RT-PCR technique (Fig. 4).

### CTC-positive rates are decreased after neoadjuvant treatment, adjuvant treatment, metastatic treatment and combination therapy, but not after surgery

**Figure 5:**
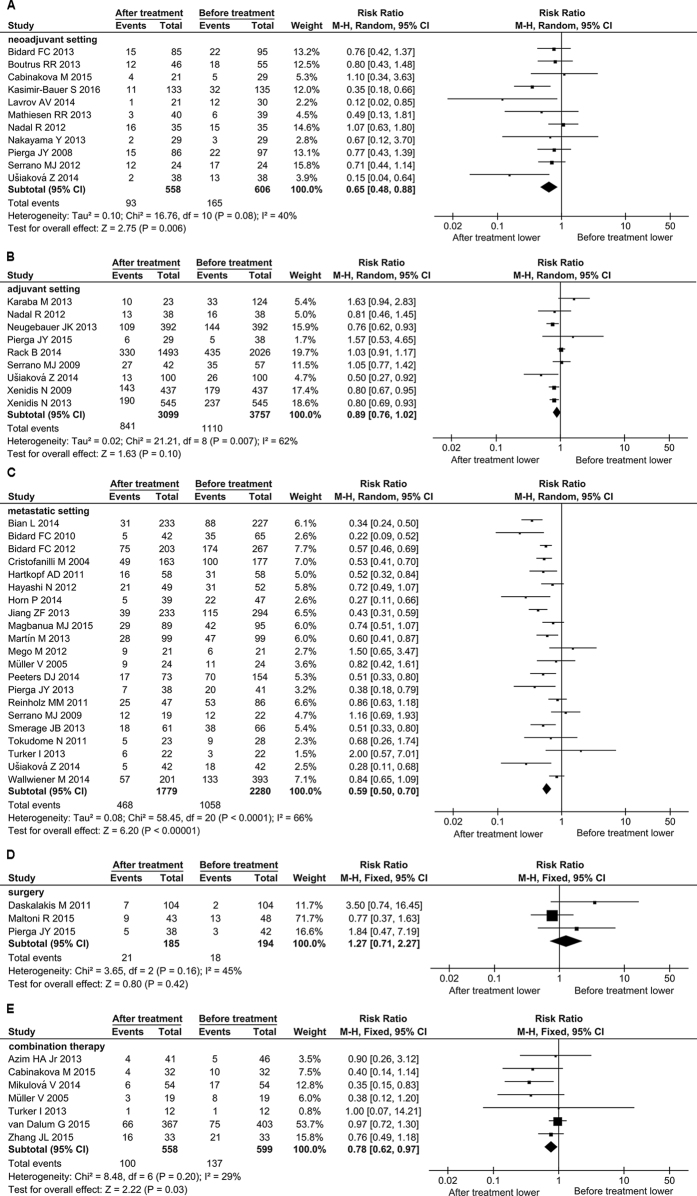
Forest plot for the comparison of CTC-positive rate before and after treatment: subgroup analysis of different therapy strategies. The results of neoadjuvant setting (**A**), adjuvant setting (**B**), metastatic setting (**C**), surgery (**D**) and combination therapy (**E**) are shown, respectively. The center of black diamond and its extremities indicate the pooled risk ratio and 95% confidential interval.

Clinically, many therapeutic methods are employed in treatment of BC. The therapeutic methods involved in the pooled 50 studies could be roughly classified into neoadjuvant setting, adjuvant setting, metastatic setting, surgery and combination therapy. To clarify the efficacy of various therapies on decreasing CTC-positive rate, we performed a subgroup analysis. Compared to pre-treatment, CTC-positive rate were decreased after treatment in the neoadjuvant setting (RR = 0.65, 95% CI: 0.48 to 0.88, *P* = 0.006; I^2^ = 40%, *P* = 0.08), and adjuvant setting (RR = 0.89, 95% CI: 0.76 to 1.02, *P* = 0.10; I^2^ = 62%, *P* = 0.007), the metastatic setting (RR = 0.59, 95% CI: 0.50 to 0.70, *P* < 0.00001; I^2^ = 66%, *P* < 0.0001) and the combination therapy (RR = 0.78, 95% CI: 0.62 to 0.97, *P* = 0.03; I^2^ = 29%, *P* = 0.20), but not in the surgery (RR = 1.27, 95% CI: 0.71 to 2.27, *P* = 0.42; I^2^ = 45%, *P* = 0.16) (Fig. 5). These results indicate that surgery as a local treatment can not eliminate CTCs timely, because CTCs can survive in peripheral blood for a certain amount of time^58^, suggesting that patients with CTC positive should be further treated with other therapies after surgery, in order to decrease the risk of metastasis and recurrence.

### CTC-positive rates are decreased after therapies in the HER2 -positive or -negative patients, but not in the triple-negative patients

**Figure 6:**
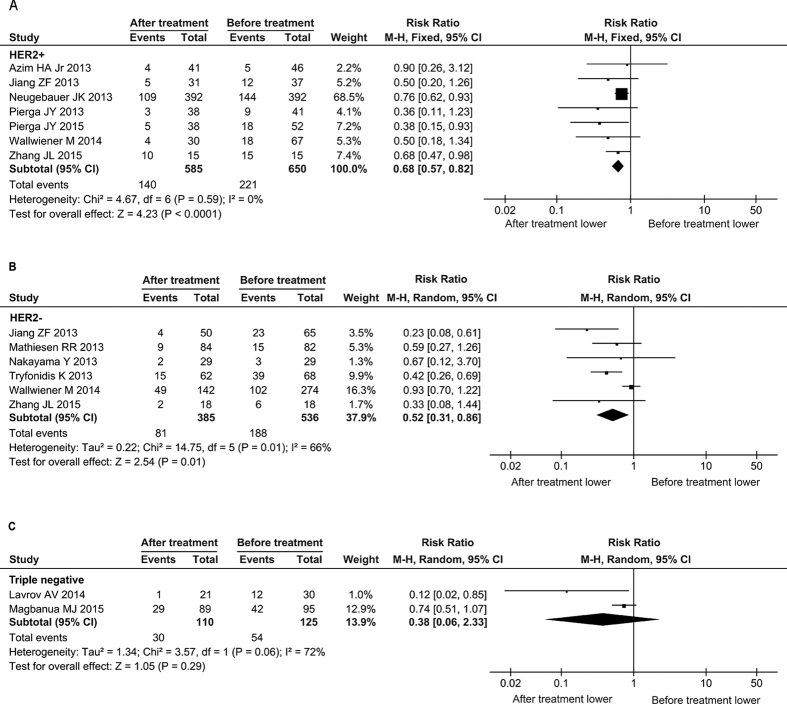
Forest plot for the comparison of CTC-positive rate before and after treatment: subgroup analysis of different molecular subtypes. The results of HER2-positive subtype (**A**) HER2-negative subtype (**B**) and triple-negative subtype (**C**) are shown, respectively. The black diamond and its extremities indicate the pooled risk ratio center and 95% confidential interval.

Currently, the clinical management of breast cancer mainly relies on the molecular subtypes based on the expression of estrogen receptor, progesterone receptor and HER2 in primary tumors. It is well known that different molecular subtypes of BC are associated with distinct malignant nature and drug response. Therefore, we further assessed the effects of therapies on reduction of CTCs in different subgroups, including HER2-positive, HER2-negative and triple-negative BC. Compared to pre-therapy, CTC-positive rates were significantly decreased after treatment in HER2-positive patients (RR = 0.68, 95% CI: 0.57 to 0.82, *P* < 0.0001; I^2^ = 0%, *P* = 0.59) and HER2-negative patients (RR = 0.52, 95% CI: 0.31 to 0.86, *P* = 0.01; I^2^ = 66%, *P* = 0.01), but not in the triple-negative ones (RR = 0.38, 95% CI: 0.06 to 2.33, *P* = 0.29; I^2^ = 72%, *P* = 0.06) (Fig. 6). These results indicate that different molecular subtypes of BC affect the efficacy of therapeutics on reducing CTCs. The poor reduction of CTC-positive rate in triple-negative BC is consistent with clinical outcome, implying that current therapies should be further optimized and the new therapeutic methods should be developed for this specific molecular subtype.

#### Correlation between status of CTCs after treatment and prognosis of the patients

**Figure 7:**
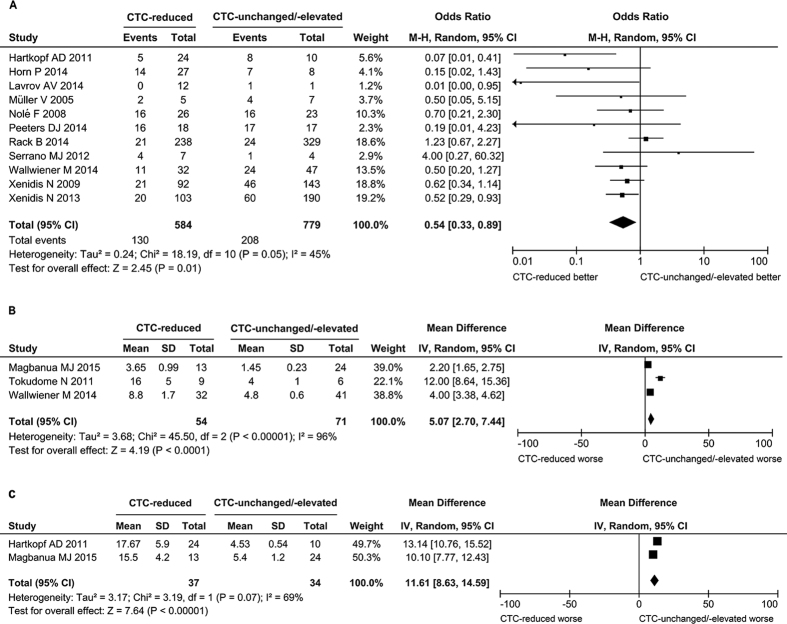
Forest plot for the comparison of prognosis between the CTC-reduced patients and those without CTC-unchanged or -elevated. The diamond indicated the odds ratio of disease progression (**A**) the difference of progression-free survival (**B**) or overall survival period (**C**). The centers of the diamonds indicated the pooled odds ratio (**A**) or the mean difference (**B**,**C**) and the extremities indicated the 95% confidential interval.

**Figure 8:**
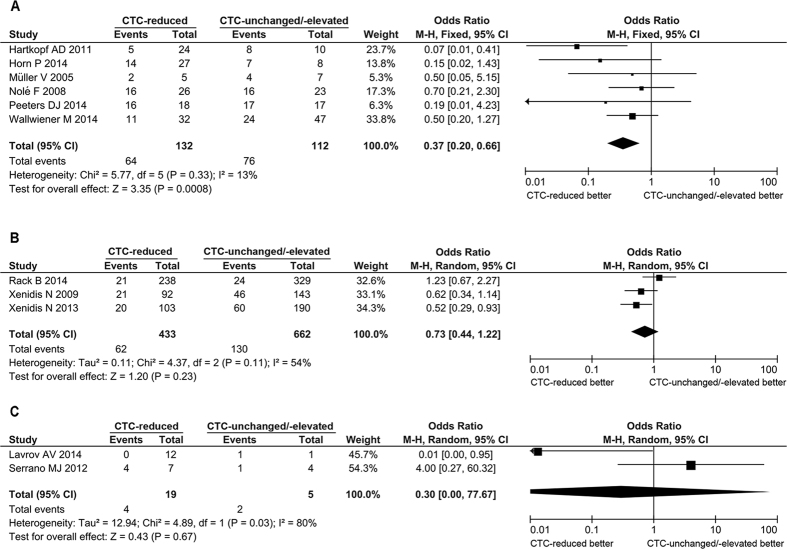
Forest plot for the comparison of prognosis between the CTC-reduced patients and those without CTC-unchanged or –elevated: subgroup analysis of disease progression. The diamond indicated the odds ratio of disease progression in the metastatic setting (**A**), the adjuvant setting (**B**) or the neoadjuvant setting (**C**). The centers of the diamonds indicated the pooled odds ratio and the extremities indicated the 95% confidential interval.

Because CTCs are shed from the primary tumor and serve as the metastatic precursors, the changes of CTC status after therapies may associate with the risk of metastasis as well as the outcome of patients. We compared the prognosis of the CTC-reduced patients with that of the CTC-unchanged or -elevated patients after treatment. The overall survival (OS) of patients after treatment was available in 2 studies (71 cases), in both of which the patients received metastatic setting. The CTC-reduced patients had a longer overall survival period compared to the CTC-unchanged or -elevated patients (mean difference = 11.61 months, 95% CI: 8.63 to 14.59, *P* < 0.00001; I^2^ = 69%, *P* = 0.07) (Fig. 7). The progression-free survival (PFS) of patients after treatment were available in 3 studies (125 patients), in which all the patients received metastatic setting. The CTC-reduced patients had a longer PFS than the CTC-unchanged or -elevated patients (mean difference = 5.07 months, 95% CI: 2.70 to 7.44, *P* < 0.0001; I^2^ = 96%, *P* < 0.00001) (Fig. 7). The disease progression of patients after treatments was available in 11 studies with 1363 patients. A significantly lower probability of disease progression was observed in the CTC-reduced patients (OR = 0.54, 95% CI: 0.33 to 0.89, *P* = 0.01; I^2^ = 45%, *P* = 0.05) (Fig. 7). The 11 studies could be divided into 3 subgroups, namely metastatic setting (6 studies with 244 patients), adjuvant setting (3 studies with 1095 patients), neoadjuvant setting (2 studies with 24 patients). Significantly lower probability of disease progression in CTC-reduced patients was observed in the metastatic setting subgroup (OR = 0.37, 95% CI: 0.20 to 0.66, *P* = 0.0008; I^2^ = 13%, *P* = 0.33) (Fig. 8). But there was no significant difference of probability of disease progression in patients with or without CTC reduction in the adjuvant setting subgroup (OR = 0.73, 95% CI: 0.44 to 1.22, *P* = 0.23; I^2^ = 54%, *P* = 0.11) and in the neoadjuvant setting subgroup (OR = 0.30, 95% CI: <0.01 to 77.67, *P* = 0.67; I^2^ = 80%, *P* = 0.03) (Fig. 8). There was a significant heterogeneity in the adjuvant-setting subgroup. When a study (Rack B 2014) was excluded, the heterogeneity was relieved (*P* = 0.68, I^2^ = 0%) and the correlation of CTC status and disease progression became significant (OR = 0.57, 95%CI: 0.37 to 0.86, *P* = 0.008 by fixed-effect model). In the neoadjuvant setting subgroup, the sample size was extremely small, implying that did not have adequate statistic power. The results showed that the reduction of CTCs was significantly associated with decreased probability of disease progression, increased overall survival and progression-free survival period.

### Sensitivity Analysis and Publication Bias

**Figure 9:**
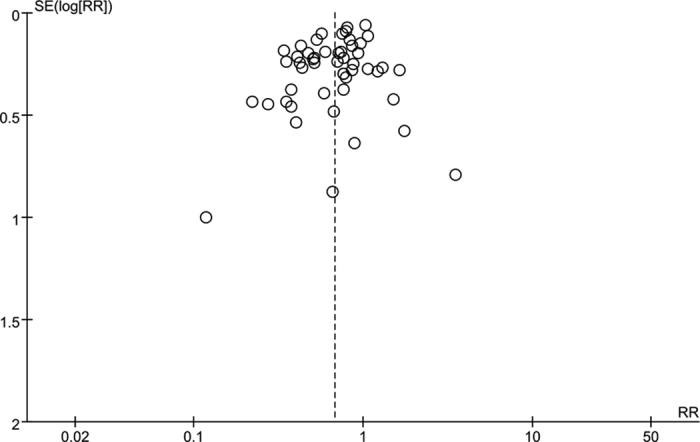
Funnel plot for the studies included for comparison of CTC-positive rate before and after treatment.

Among the 50 studies included for the pooled RR estimation, no single one contributed substantial influence. When we analyzed the change of CTC counts post-treatments with 7 studies reported CTC status both in CTC-positive rate and CTC counts, the sensitivity analysis was tested by removal of each study. One of them (Bidard FC 2012) was found to substantially affect the heterogeneity and the significance of overall effect: when it was excluded, the heterogeneity was relieved (P = 0.69, I^2^ = 0%) and the change of CTC counts after treatment became significant (mean difference = −0.94, 95%CI: −1.49 to −0.38, *P* = 0.0010 by fixed-effect model). In the adjuvant-setting subgroup analysis of disease progression (Fig. 8), one of the three recruited studies (Rack B 2014) was found to substantially affect the heterogeneity and the significance of overall effect: when it was excluded, the heterogeneity was relieved (*P* = 0.68, I^2^ = 0%) and the correlation of CTC status and disease progression became significant (OR = 0.57, 95%CI: 0.37 to 0.86, *P* = 0.008 by fixed-effect model). No substantial publication bias was found according to the Funnel plot (Fig. 9).

## Discussion

It has been well known that even localized tumors without clinically apparent metastasis give rise to CTCs. Because generation of CTCs is an indispensable step of the metastatic process of tumors, the promising application as a noninvasive blood biomarker in prognosis and response to therapy are very attractive. Although the actual utility of CTCs remains largely academic^59^, many studies have reported the detection of CTCs to facilitate early diagnosis of relapse or metastasis and improve the treatment decisions. In present meta-analysis, we analyzed the changes of CTC status after therapies compared to that of before therapies in pooled 50 studies with 6712 BC patients, and demonstrated that CTC status was a useful indicator to monitor the treatment response, and predict the outcome of patients.

The actual application of CTCs in clinical setting relies on the progression of detection technologies. In the past decades, a number of technically diverse platforms have been developed for CTC assay^60^. However, for any technology to be used in the clinic, demonstration of analytic validity, clinical validity, and clinical utility is required^60^. Up to now, the only system approved by the Food and Drug Administration (FDA) as an aid in monitoring patients with metastatic breast, colorectal, or prostate cancer is CellSearch^®^ (Veridex, Raritan, NJ, USA). Also, CellSearch^®^ is the only semi-automated system and has contributed considerably to the development of standards for CTC enumeration. Nevertheless, there are disadvantages to be perfect for it. Its enrichment/capture technology is based on epithelial marker EpCAM, which is usually with low sensitivity and efficiency, due to the lost expression of EpCAM in CTCs by EMT process^61^,^62^. In our meta-analysis, other CTC detection methods are employed in some studies, including RT-PCR, which determined the CTC status by detecting the mRNA expression of epithelial markers, such as EpCAM or CKs. There is a probability that the CTC detection methods based on different labels or rationales would find different counts of CTCs in the same individual patients. Given the CTC rarity, especially in non-metastatic breast cancer, continuous training and central image review is required in order to gain best inter-reader agreement. A recent study evaluated the inter-reader agreement in 22 readers from 15 academic laboratories and 8 readers from two Veridex laboratories with non-metastatic (M0) and metastatic (M1) breast cancer samples. For CTC definition (No CTC vs. CTC), the median agreement between academic readers and VC was 92% (range 69 to 97%) with a median κ of 0.83 (range: 0.37 to 0.93). The inter-reader agreement for CTC definition was high. Reduced agreement was observed in M0 patients with low CTC counts^63^. In addition, the inconsistence of the cut-off value to determine CTC-positive amongst studies would be a limitation for actual application of CTCs in clinical setting (Fig. 4). Standard or uniform protocol for CTC measurement would be required before this indicator could be clinically adopted.

There were several limitations in the present research. First, most of studies included in this meta-analysis were consisted of patients receiving certain therapy alone without an appropriate negative control. Second, some studies with substantial sample heterogeneities, which were caused by the complexity of patient characteristics (race, age), therapeutic details (drug, dose, treatment periodicity, etc.) or other factors, were excluded in our subgroup analyses. Third, the sample sizes of some subgroups were relatively small, which might affect the detection of potential difference. Fourth, CTCs were thought to be a set of cells with different characteristics, so it would be more meaningful to investigate the correlation of change of the CTC subpopulations after treatment with patient prognosis. However, so far few data are available to perform an analysis.

In summary, the present meta-analysis demonstrated that the status of CTCs is a useful indicator of the efficacy of therapies for BC, which may help clinicians make a decision for further personalized therapy of patients. However, it is on the way for application of CTCs in clinical setting because there are still challenges presented in analytic validity, clinical validity, and clinical utility of CTCs.

## Methods

### Search strategy

A comprehensive literary search for potential studies was searched in June 2016 without time or language restrictions. The electronic databases include PubMed, EMBase and Cochrane library. The keywords and MeSH terms were variably combined: “circulating tumor cell (s)”, “breast cancer”, “therapy”. The search strategy was intended to exclude reviews, comments, letters and editorials, which have irrelevant study data, by screening the titles and abstracts of publications.

### Eligibility criteria

Inclusion criteria: (i) enrolled patients with BC were pathologically diagnosed; (ii) CTCs were detected by any method, including cell capture and quantitative PCR; (iii) the patients’ CTC status both pre- and post-therapy was reported.

Exclusion criteria: (i) cell-line experiments or animal models; (ii) a small size <20 patients; (iii) when information of the same patients was reported in different studies, only the latest and most informative one was included; (iv) CTC status was reported pre- or post-therapy alone; (v) CTC status pre- and post-therapy was reported in different cohort of patients; (vi) Studies which reported CTCs only in continuous style were excluded, as their results could not be integrated with the majority of studies which reported the results in dichotomous pattern (positive vs. negative).

### Data extraction

Studies were reviewed and extracted independently by two reviewers (WT Yan and Q Chen). The primary data of the included studies were following: the general information (i.e., the first author, the year of publication, the nationality of studies), sample size, the patients’ characteristics (i.e., ages, tumor stage, molecular subtypes of tumor), assessment of CTCs (i.e., methods of CTC detection, blood volume, the cut-off value of CTCs, the count of CTCs and/or the positive rate of CTCs) and the type of the treatments. If CTCs at more than one phase of follow ups were reported in a study, the latest phase with follow-up rate ≥75% was chosen; if no phase met the criteria, the earliest phase was chosen. The prognosis (progression-free survival, overall survival) was also extracted for assessing prognostic value of reducing CTCs.

### Quality assessment of primary studies

The quality of each included study was evaluated by a scale based on the Newcastle–Ottawa Quality Assessment Scale.

### Statistical analysis

The pooled RR (relative risk) and mean difference were calculated to analyze the difference of CTCs between pre-therapy and post-therapy by fixed model or random-effects model according to the heterogeneity of the studies, which was estimated by the Cochran’s Q test and the I^2^ index (P value < 0.10 or I^2^ over 50% was define as substantial heterogeneity). RR less than 1 or mean difference less than 0 indicated declined CTCs in the peripheral blood. Pooled OR (odds ratio) and mean difference were calculated to analyze the difference of progression between the patients with different dynamic conditions of CTCs (decreasing vs. increasing or persistent elevated). Subgroup analyses were performed for CTC determination methods, treatments and molecular subtypes of primary tumor as long as two or more studies were available to be included. Forest plot was used to illustrate the pooled RR mean difference. All analyses were run by Review Manager Version 5.3 (Cochrane Collaboration, Copenhagen, Denmark).

### Sensitivity analysis and estimation of publication bias

To evaluate the influence of individual study on the pooled RR or mean difference, sensitivity analysis was performed by removing each eligible study separately. Funnel plot developed by Begg was used to detect potential publication bias which might affect the validity of the results.

## Additional Information

**How to cite this article:** Yan, W.-T. *et al*. Circulating tumor cell status monitors the treatment responses in breast cancer patients: a meta-analysis. *Sci. Rep.* **7**, 43464; doi: 10.1038/srep43464 (2017).

**Publisher's note:** Springer Nature remains neutral with regard to jurisdictional claims in published maps and institutional affiliations.

## Supplementary Material

Supplementary Tables and Figures
