# Supplementary material for: Circulating tumor cell status monitors the treatment responses in breast cancer patients: a meta-analysis
Source: Sci Rep. 2017 Mar 24;7:43464. doi: 10.1038/srep43464 (PMC5364512; doi:10.1038/srep43464)
Supplement: Supplementary Tables and Figures [file srep43464-s1.pdf]

Circulating tumor cell status monitors the treatment responses in breast cancer patients: a meta-analysis

Wen-ting Yan[1], Xiang Cui[1], Qing Chen[2], Ya-fei Li[3],You-hong Cui[4], Yan Wang[4], Jun Jiang[1*]

[1]Breast Disease Center, Southwest Hospital, Third Military Medical University, Chongqing 400038, China

[2]Institute of Toxicology, College of Preventive Medicine, Third Military Medical University, Chongqing 400038, China

[3]Department of Epidemiology, College of Preventive Medicine, Third Military Medical University, Chongqing 400038, China

[4]Institute of Pathology and Southwest Cancer Center, Southwest Hospital, Third Military Medical University Chongqing 400038, China

*corresponding author: jcbd@medmail.com.cn.

Supplementary tables

Table S1. Characteristics of the involved studies

Study	Sample size	Patient characteristics		CTC detection				Therapy
		Age, years	Molecular subtype	Blood volume, mL	Detection method	Result presentation	CTC-positive Cut-off value	
Kasimir-Bauer S 2016 (Germany)	135	51 (median)	-	5	RT-PCR	Positive rate	Transcript >0.15 ng/μL	N
Cabinakova M 2015(Czech)	50	37 (33.75-40.50)	-	7	AdnaTest Breast Cancer™	Positive rate	≥1 CTC/7 mL	C, N
Magbanua MJ 2015(USA)	102	49 (33-71) , n=31<br>52 (28-83) , n=71	Triple negative	7.5-10	CellSearch, IE/FC	Positive rate	≥5 CTC/7.5 mL	M
Maltoni R 2015(Italy)	48	NA	NA	18-20	DEPArray	Positive rate	≥ 1 CTC/7.5 mL<br>≥ 2 CTCs/7.5 mL<br>≥ 3 CTCs/7.5 mL	S
Pierga JY 2015(France)	52	50.6	HER2+	7.5	CellSearch	Positive rate	≥ 1 CTC/7.5 mL	S, A
van Dalum G 2015(Italy)	403	NA	NA	30	CellSearch	Positive rate	≥ 1 CTC/30 mL	C
Wang HY 2015(Korea)	221	49. 8 ± 9.9	NA	7.5	CircleGen CTC RT-qDx assay	Positive rate	-	C or N or M
Zhang JL 2015(China)	33	NA	HER2+/-	NA	RT-PCR	Positive rate	-	C

Barnadas A 2014(Spain)	234	59.8 ± 13.3	-	7.5	CellSearch	Positive rate	≥ 5 CTCs/7.5 mL	Bisphosphonates
Bian L 2014(China)	300	50 (29, 69)	-	7.5	CellSearch	Positive rate	≥ 5 CTCs/7.5 mL	M
Horn P 2014(Denmark)	47	59.5 (30,81)	-	7.5	CellSearch	Positive rate<br>Counts	≥ 5 CTCs/7.5 mL	M
Lavrov AV 2014(Russia)	30	44 (28-72)	Triple negative	NA	Adnagen	Positive rate	-	N
Mikulová V 2014(Czech)	54	36 (22-72)	-	5	AdnaTest Breast Cancer™	Positive rate	≥ 2 CTCs/5 mL	C
Peeters DJ 2014(Belgium)	154	62.1 (32.9-90.8)	-	NA	CellSearch	Positive rate	≥ 5 CTCs/7.5 mL	M
Rack B (plus Jaeger BA 2014) 2014(Germany)	2026	53.0 (21-85)	-	30	CellSearch	Positive rate<br>Counts	≥ 1 CTC/30 mL<br>≥ 2 CTCs/30 mL<br>≥ 5 CTCs/30 mL<br>≥ 10 CTCs/30 mL	A
Ušiaková Z 2014(Czech)	179	49.1 (mean)	-	5	RT-PCR	Positive rate	-	N, A, M
Wallwiener M 2014(Germany)	393	59 (29-89)	HER2+/- triple-negative	7.5	CellSearch	Positive rate	≥ 5 CTCs/7.5 mL	M
Azim HA Jr 2013(Belgium)	55	CTC-positive:<br>55 (41-72) , n=11<br>CTC-negative:<br>52 (25-73) , n=40	HER2+	22.5	CellSearch	Positive rate	≥ 1 CTC /22.5 mL	C
Bidard FC 2013(France)	115	47 (29-65)	NA	7.5	Cellsearch	Positive rate	≥ 1 CTC/7.5 mL	N

Boutrus RR 2013(USA)	55	48 (41-54)	NA	8	RT-PCR	Positive rate	NA	N
Jiang ZF 2013(China)	294	49.4 ± 9.4	HER2+<br>Triple negative	10	CellSearch	Positive rate	≥ 5 CTCs/7.5 mL	M
Karaba M 2013(Slovakia)	124	NA	-	NA	RT-PCR	Positive rate	NA	A
Martín M 2013(Spain)	117	61 (33-88)	-	7.5	CellSearch	Positive rate	≥ 5 CTCs/7.5 mL	M
Mathiesen RR 2013(Norway)	90	NA	HER2-	22.5	CellSearch	Positive rate	≥ 1 CTC/22.5 mL	N
Nakayama Y 2013(Japan)	29	NA	HER2-	NA	NA	Positive rate	≥ 1 CTC	N
Neugebauer JK 2013(Germany)	392	NA	HER2+	23	CellSearch	Positive rate	≥ 1 CTC/23 mL	A
Pierga JY 2013(France)	45	56 (35-79)	HER2+	7.5	CellSearch	Positive rate	≥ 1 CTC/7.5 mL<br>≥ 5 CTCs/7.5 mL	M
Roop RP 2013(USA)	24	50.7 (mean)	-	7.5	CellSearch	Positive rate	≥ 1 CTCs/7.5 mL	Platelet function inhibition
Smerage JB 2013(USA)	83	NA	-	7.5	CellSearch	Positive rate	≥ 5 CTCs/7.5 mL	M
Tryfonidis K 2013(Greece)	83	62.0 (23-75)	HER2-	7.5	CellSearch	Positive rate	≥ 1 CTC/7.5 mL<br>≥ 5 CTCs/7.5 mL	A or M
Turker I 2013(Turkey)	34	early stage<br>48.0, n=12 (median)<br>Metastatic<br>52.5, n=22 (median)	-	7.5	CellSearch	Positive rate	≥ 5 CTCs/7.5 mL	M, C

Xenidis N 2013(Greece)	545	Taxane-free: 54 (30-75)<br>Taxane-based: 53 (26-76)	-	20	RT–PCR	Positive rate	≥0.6 MCF-7 equivalents/5μg total RNA	A
Aurilio G 2012(Italy)	56	NA	NA	NA	CellSearch	Positive rate	≥ 5 CTCs/7.5 mL	A or M
Bidard FC 2012(France)	267	57 (median)	-	7.5	CellSearch	Positive rate<br>Counts	≥ 1 CTC/7.5 mL<br>≥ 5 CTCs/7.5 mL	M
Hayashi N 2012(Japan)	52	54.1 (32-74)	-	7.5	CellSearch	Positive rate	≥ 1 CTC/7.5 mL<br>≥ 5 CTCs/7.5 mL	M
Mego M 2012(USA)	21	44 (30-60)	-	7.5	CellSearch	Positive rate	≥ 1 CTC/7.5 mL<br>≥ 5 CTCs/7.5 mL	M
Nadal R 2012(Spain)	98	65.3% (64/98)>50	-	30	Immunomagnetic	Positive rate	≥ 1 CTC/30 mL	N, A
Serrano MJ 2012(Spain)	24	26-71 (range)	-	10	Immunomagnetic	Positive rate<br>Counts	≥ 1 CTC/10 mL	N
Daskalakis M 2011(Greece)	104	56. 8 ± 13.1	-	20	RT-PCR	Positive rate	≥0.6 MCF-7 equivalents/5μg total RNA	S
Hartkopf AD 2011(Germany)	58	NA	-	7.5	CellSearch	Positive rate	≥ 5 CTCs/7.5 mL	M
Reinholz MM 2011(USA)	86	NA	-	10	RosetteSep Human CD45 Depletion Cocktail	Positive rate	CK19mRNA: 2–△△Cq≥2<br>MGB1: 2–△△Cq≥2	M
Tokudome N 2011(Japan)	28	54.5 (mean)	-	10	CellSearch	Positive rate	≥ 5 CTCs/7.5 mL	M
Bidard FC 2010(France)	67	83.6% (56/67) ≥45	-	7.5	CellSearch	Positive rate<br>Counts	≥ 5 CTCs/7.5 mL	M

Cristofanilli M 2009(USA)	102	NA	NA	7.5	CellSearch	Positive rate	≥ 5 CTCs/7.5 mL	NA
Serrano MJ 2009(Spain)	71	55 (26-77)	-	10	Carcinoma Cell Enrichment and Detection kit	Positive rate	≥1 CTC/10 mL	A, M
Xenidis N 2009(Greece)	437	54.0 (26-87)	-	10	RT-PCR	Positive rate Counts	≥0.6 MCF-7 equivalents/5μg total RNA	A
Nole´ F 2008(Italy)	80	55 (32-77)	-	10	CellSearch	Positive rate	≥ 5 CTCs/7.5 mL	NA
Pierga JY 2008(France)	118	47 (29-65)	-	7.5	CellSearch	Positive rate Counts	≥ 1 CTC/7.5 mL	N
Müller V 2005(Germany)	43	NA	-	20	OncoQuick	Positive rate	≥1 CTC	M; C
Cristofanilli M 2004(USA)	177	58.0 ± 13.4	-	10	CellSearch	Positive rate	≥ 5 CTCs/7.5 mL	M

C, combination therapy; N, neoadjuvant setting; M, metastatic setting; S, surgery; A, adjuvant setting; NA, not available.
Age was represented as “mean ± standard deviation” or “median (interquartile range)” if not specifically indicated.

Table S2. Estimation of the study quality according to the Newcastle-Ottawa Quality Assessment Scale (cohort studies)

Study	Representativeness of the exposed cohort	Selection of the non-exposed cohort	Ascertainment of exposure	Outcome of interest was not present at start	Comparability	Assessment of outcome	Was follow-up long enough	Adequacy of follow up of cohorts	Total
Kasimir-Bauer S 2016 (Germany)	1	1	1	1	2	1	1	1	9
Cabinakova M 2015(Czech)	1	1	1	1	2	1	1	0	8
Magbanua MJ 2015(USA)	1	1	1	1	2	1	1	1	9
Maltoni R 2015(Italy)	1	1	1	1	2	1	1	1	9
Pierga JY 2015(France)	0	1	1	1	2	1	1	0	7
van Dalum G 2015(Italy)	1	1	1	1	2	1	1	1	9
Wang HY 2015(Korea)	1	1	0	1	2	1	1	1	8
Zhang JL 2015(China)	1	1	1	1	2	1	1	1	9
Barnadas A 2014(Spain)	1	1	1	1	2	1	1	0	8

Bian L 2014(China)	1	1	1	1	2	1	1	1	9
Horn P 2014(Denmark)	1	1	1	1	2	1	1	1	9
Lavrov AV 2014(Russia)	1	1	1	1	2	1	1	0	8
Mikulová V 2014(Czech)	1	1	1	1	2	1	1	1	9
Peeters DJ 2014(Belgium)	1	1	1	1	2	1	1	0	8
Rack B (plus Jaeger BA 2014) 2014(Germany)	1	1	1	1	2	1	1	0	8
Ušiaková Z 2014(Czech)	1	1	1	1	2	1	1	1	9
Wallwiener M 2014(Germany)	1	1	1	1	2	1	1	0	8
Azim HA Jr 2013(Belgium)	1	1	1	1	2	1	1	1	9
Bidard FC 2013(France)	1	1	1	1	2	1	1	1	9
Boutrus RR 2013(USA)	1	1	1	1	2	1	1	1	9
Jiang ZF 2013(China)	1	1	1	1	2	1	1	1	9
Karaba M 2013(Slovakia)	0	1	1	1	2	1	1	0	7

Martín M 2013(Spain)	1	1	1	1	2	1	1	1	9
Mathiesen RR 2013(Norway)	0	1	1	1	2	1	1	1	8
Nakayama Y 2013(Japan)	0	1	1	1	2	1	1	1	8
Neugebauer JK 2013(Germany)	1	1	1	1	2	1	1	1	9
Pierga JY 2013(France)	1	1	1	1	2	1	1	1	9
Roop RP 2013(USA)	1	1	1	1	2	1	1	1	9
Smerage JB 2013(USA)	1	1	1	1	2	1	1	1	9
Tryfonidis K 2013(Greece)	1	1	1	1	2	1	1	0	8
Turker I 2013(Turkey)	1	1	1	1	2	1	1	1	9
Wang H 2013(USA)	1	1	1	1	2	1	1	0	8
Xenidis N 2013(Greece)	1	1	1	1	2	1	1	1	9
Aurilio G 2012(Italy)	0	1	1	1	2	1	1	1	8
Bidard FC 2012(France)	1	1	1	1	2	1	1	1	9

Hayashi N 2012(Japan)	1	1	1	1	2	1	1	1	9
Mego M 2012(USA)	1	1	1	1	2	1	1	1	9
Nadal R 2012(Spain)	1	1	1	1	2	1	1	0	8
Serrano MJ 2012(Spain)	1	1	1	1	2	1	1	1	9
Daskalakis M 2011(Greece)	1	1	1	1	2	1	1	1	9
Hartkopf AD 2011(Germany)	1	1	1	1	2	1	1	1	9
Reinholz MM 2011(USA)	1	1	1	1	2	1	1	0	8
Tokudome N 2011(Japan)	1	1	1	1	2	1	1	1	9
Bidard FC 2010(France)	1	1	1	1	2	1	1	0	8
Cristofanilli M 2009(USA)	0	1	1	1	2	1	1	1	8
Serrano MJ 2009(Spain)	0	1	1	1	2	1	1	1	8
Xenidis N 2009(Greece)	1	1	1	1	2	1	1	1	9
Nole´ F 2008(Italy)	1	1	1	1	2	1	1	1	9

Pierga JY 2008(France)	1	1	1	1	2	1	1	1	9
Müller V 2005(Germany)	1	1	1	1	2	1	1	1	9
Cristofanilli M 2004(USA)	1	1	1	1	2	1	1	1	9

Study	After treatment Events	After treatment Total	Before treatment Events	Before treatment Total	Weight	Risk Ratio M-H, Random, 95% CI
Bidard FC 2010	5	42	35	65	7.6%	0.22 [0.09, 0.52]
Bidard FC 2012	75	203	174	267	19.4%	0.57 [0.46, 0.69]
Horn P 2014	5	39	22	47	7.4%	0.27 [0.11, 0.66]
Pierga JY 2008	15	86	22	97	11.5%	0.77 [0.43, 1.39]
Rack B 2014	330	1493	435	2026	20.5%	1.03 [0.91, 1.17]
Serrano MJ 2012	12	24	17	24	13.7%	0.71 [0.44, 1.14]
Xenidis N 2009	143	437	179	437	19.8%	0.80 [0.67, 0.95]
**Subtotal (95% CI)**		**2324**		**2963**	**100.0%**	**0.65 [0.48, 0.87]**
Total events	585		884			

Heterogeneity: Tau² = 0.11; Chi² = 41.18, df = 6 (P < 0.00001); I² = 85%

Test for overall effect: Z = 2.92 (P = 0.004)

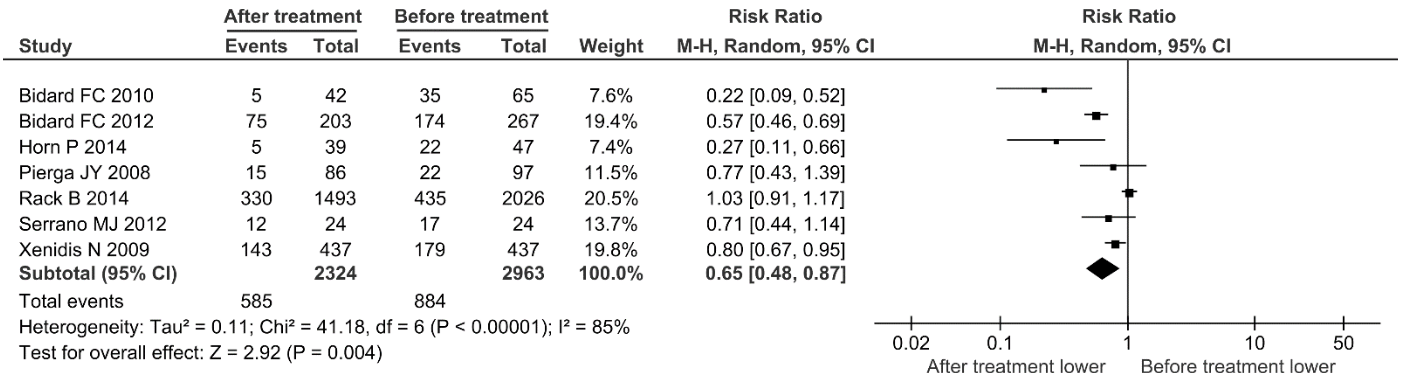

Figure S1. Forest plot for the comparison of CTC-positive rate before and after treatment: subgroup analysis in the seven studies which also supplied CTC data in counts. The center of black diamond and its extremities indicate the pooled risk ratio and 95% confidential interval.
